# From Biomass to Fuel Blendstocks via Catalytic Fast Pyrolysis and Hydrotreating: An Evaluation of Carbon Efficiency and Fuel Properties for Three Pathways

**DOI:** 10.1021/acs.energyfuels.3c03239

**Published:** 2023-11-29

**Authors:** Kristiina Iisa, Calvin Mukarakate, Richard J. French, Foster A. Agblevor, Daniel M. Santosa, Huamin Wang, A. Nolan Wilson, Earl Christensen, Michael B. Griffin, Joshua A. Schaidle

**Affiliations:** † National Renewable Energy Laboratory, Golden, Colorado 80403, United States; ‡ Utah State University, Logan, Utah 84322, United States; § Pacific Northwest National Laboratory, Richland, Washington 99354, United States

## Abstract

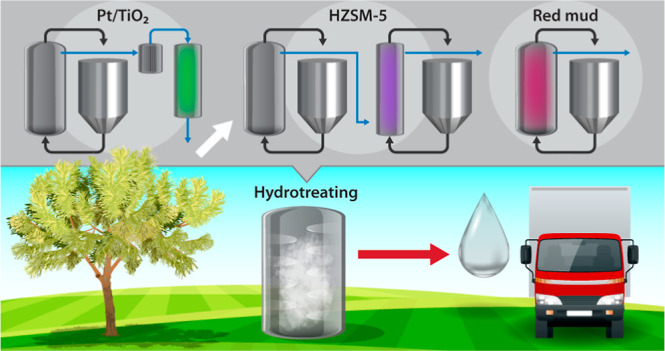

Biomass was upgraded to fuel blendstocks via catalytic fast pyrolysis (CFP) followed by hydrotreating using three approaches: ex situ CFP with a zeolite catalyst (HZSM-5), ex situ CFP with a hydrodeoxygenation catalyst (Pt/TiO_2_) and cofed hydrogen, and in situ CFP with a low-cost mixed metal oxide catalyst (red mud). Each approach was evaluated using a common pine feedstock and the same hydrotreating procedure. The oxygen contents in the CFP oils ranged from 17 to 28 wt % on a dry basis, and the carbon efficiencies for the CFP processes were in the range of 28–38%. The residual oxygen was reduced to <1 wt % during hydrotreating, which was operated for 104–140 h for each CFP oil without plugging issues. The hydrotreating carbon efficiencies were 81–93%. The CFP pathway with the hydrodeoxygenation catalyst gave the highest overall carbon efficiency from biomass to fuel blendstocks (34%) but, at the same time, also the highest cumulative hydrogen consumption during CFP and hydrotreating. The zeolite pathway produced the largest fraction boiling in the gasoline range and the highest estimated octane number due to the high aromatic content in that CFP oil. The in situ red mud pathway produced the largest fraction of diesel-range products with the highest derived cetane number. However, advances in the CFP and hydrotreating process are required to improve the fuel blendstock properties for all pathways.

## Introduction

The conversion of biomass to hydrocarbon fuels offers an approach to reduce greenhouse gas emissions and introduce renewable feedstocks into the transportation sector. Catalytic fast pyrolysis (CFP) is a promising method for converting lignocellulosic biomass into hydrocarbon fuel precursors and blendstocks.^1,2^ In this process, biomass is thermally deconstructed via fast pyrolysis, and the resulting vapors are catalytically upgraded prior to condensation to improve the quality and stability of the oil product. The oxygen remaining in the CFP oil can be subsequently removed by hydrotreating at high pressures in the presence of a catalyst in a process similar to petroleum hydroprocessing, and the hydrotreated products can be fractionated by distillation into fuel blendstocks.

CFP may take place in either an in situ or ex situ configuration.^1,3−5^ During in situ CFP, the catalyst is placed directly in the pyrolysis reactor, while in ex situ CFP, the pyrolysis products are catalytically upgraded in a separate downstream reactor. The in situ configuration offers a simpler process with fewer reactors, but the catalyst is in contact with the biomass, char, and ash, which may lead to irreversible catalyst deactivation by biomass contaminants.^6−8^ The ex situ configuration has a higher capital cost due to the added upgrading reactor, but the catalyst is not in direct contact with the biomass. The catalyst can be further shielded from vapor phase contaminants from the biomass by a hot gas filter.^6,7,9^ The utilization of a hot gas filter may enhance cracking reactions and has been reported to reduce fast pyrolysis oil yields by 4–30%;^9,10^ however, the impact on the yields of CFP oil is not known. The ex situ configuration also allows separate control of the pyrolysis and upgrading process.^3^

Zeolites, especially HZSM-5, are common materials employed in CFP because they are effective at deoxygenating biomass pyrolysis vapors to produce fuel-range aromatic hydrocarbons.^11−13^ HZSM-5 removes oxygen from biomass pyrolysis products via a series of decarbonylation, decarboxylation, dehydration, cracking, and coupling reactions to form light alkenes and one- and multiring aromatic hydrocarbons. Partially deoxygenated products, such as phenols and furans, are also formed during this process, and the products may also include primary pyrolysis vapors such as methoxyphenols and anhydrosugars.^14^ The proportion of aromatic hydrocarbons, partially upgraded products, and primary pyrolysis compounds in the CFP oil depends on the biomass-to-catalyst ratio used during the CFP process.^6,14−16^ The reactions that lead to the formation of aromatic hydrocarbons also lead to buildup of condensed carbon (i.e., coke) on the catalyst, which reduces oil yields and rapidly deactivates the catalyst. Additional hydrocarbon yield losses occur through the light gas formation. The deactivation caused by the carbon deposits can be reversed by thermal oxidation of the catalyst, and commercial-scale upgrading over zeolites is envisioned to take place in riser reactors with continuous regeneration of the spent catalysts.^17^ Bench- and pilot-scale experiments of biomass CFP over HZSM-5-based catalysts have shown carbon efficiencies of 21–33% for producing CFP oils with 18–24 wt % oxygen.^5,13,16,18^ While limited differences in the performance of HZSM-5 catalyst in the in situ and ex situ configuration have been found in short-term bench-scale experiments,^1,5^ longer experiments and studies of metal addition have pointed to irreversible deactivation of HZSM-5 and lower CFP oil yields caused by alkali and alkaline earth metals (e.g., Na, K, Ca) found in biomass.^8,13^ This suggests that without feed demineralization, ex situ CFP may be better suited than in situ CFP for upgrading over zeolite catalysts.

The in situ CFP process requires stable, low-cost, and robust catalysts, which are easily regenerated and resistant to mineral deposition. Gamma-alumina was demonstrated for in situ CFP at the bench and pilot scale,^19^ producing oils with 16–23 wt % oxygen but at a relatively low carbon efficiency of 12–15%. Red mud, the solid residue from the processing of bauxite to alumina using the Bayer process, is another material tested at the bench scale for in situ CFP.^20−23^ Red mud is a complex mixture of metallic oxides, such as ferric oxide, aluminum oxide, titanium dioxide, magnesium oxide, calcium oxide, silicon oxide, and other minor compounds, in addition to the residual sodium hydroxide used in the extraction process. Red mud is often considered a waste product that is readily available in ton quantities. CFP of pinyon juniper over red mud produced oils with 22–27 wt % oxygen with carbon efficiencies of up to 51%.^20,22^ The CO_2_ yields were higher than the CO yields, which is beneficial for retaining carbon in the liquid phase. The presence of both basic and acid sites in red mud enhances the catalytic activity of red mud.^1,23^ Regeneration of the catalyst by burning off coke restored the catalyst activity.^20,23^

Riser reactors and fluidized bed reactors are utilized for in situ CFP and ex situ CFP over zeolite catalysts.^16^ Another approach for CFP employs ex situ upgrading of pyrolysis vapors with a nonzeolite material in a fixed-bed reactor system.^24^ Due to reduced attrition and comparatively low catalyst replacement rates, fixed-bed reactors open opportunities to explore higher-value catalyst formulations, but they require that any catalyst regeneration be performed in the reactor. These catalysts may lead to improved organic liquid yields by promoting hydrodeoxygenation (HDO) reaction in the presence of added hydrogen, where oxygen is removed as water without breaking the C–C bonds of reactants.^24^ Recent research activities have demonstrated that bifunctional catalysts containing a combination of metallic and acidic sites are effective for deoxygenation under CFP conditions with added hydrogen.^25−33^ Among this class of materials, catalysts composed of noble metals (e.g., Pt, Pd, Ru) dispersed on reducible oxide supports (e.g., TiO_2_, ZrO_2_) have demonstrated promising performance when evaluated using model compounds.^32,34−37^ Evaluations of fuel blendstock production with ex situ fixed-bed upgrading of pine pyrolysis vapors over a Pt/TiO_2_ catalyst showed a CFP carbon efficiency of approximately 35% for oil with 16–19 wt % oxygen on dry basis.^2,38^

The production of fuel blendstocks from the CFP oils requires hydrotreating to remove the oxygen remaining in the CFP oil. Several stages of hydrotreating at increasing severity are needed for the complete deoxygenation of noncatalytic pyrolysis oils.^39,40^ The CFP process may eliminate or reduce the contents of the most reactive species in pyrolysis oils, e.g., aldehydes and anhydrosugars, and enable hydrotreating in a single stage. Single-stage hydrotreating of in situ CFP oil produced over red mud from pinyon juniper was demonstrated for over 300 h without evidence of catalyst fouling^21^ and of CFP oil from pine from ex situ fixed-bed CFP over Pt/TiO_2_ for 140 h.^2^ The carbon efficiencies for hydrotreating were high, 89–93%.^2,21^ Hydrotreating carbon efficiency decreases as the CFP oil oxygen content increases,^15^ and retaining a high content of oxygenated compounds may also necessitate a stabilizing step during hydrotreating. On the other hand, producing CFP oil with low oxygen content reduces CFP carbon efficiency;^14^ therefore, a balance between oxygen removal during the CFP and hydrotreating steps is required.^41^

The objective of the current study was to make a side-by-side comparison of the performance of different CFP approaches and to identify the targeted areas for future research and development. The chosen processes included (1) ex situ CFP over a zeolite catalyst, (2) ex situ fixed-bed CFP over a HDO catalyst with cofed H_2_, and (3) in situ CFP over a low-cost, inorganic-resistant catalyst. For the ex situ zeolite process, an HZSM-5-based catalyst was chosen, and the experiments were conducted in a combination of a fluidized-bed pyrolyzer and a fluidized-bed upgrading reactor with a continuous feed and removal of catalyst. For the ex situ HDO CFP, pyrolysis vapors from the same pyrolyzer were upgraded in a fixed-bed reactor containing Pt/TiO_2_ catalyst, as reported earlier.^2^ For the in situ case, results from fluidized bed pyrolysis in the presence of red mud catalyst were utilized, with greater details on product properties reported here, in addition to the yield data previously provided.^22^ The same feedstock (southern pine) constituted the feed in all of the processes, but the CFP conditions, including temperature and frequency of catalyst regeneration, were chosen independently for each approach. The CFP oils were hydrotreated under identical conditions in the same continuous single-stage hydrotreater. The hydrotreated oils were then fractionated into gasoline and diesel blendstocks, and the fractions were evaluated for their fuel properties.

## Experimental Section

### Materials

The biomass feedstock was loblolly pine supplied by Idaho National Laboratory. The feedstock was provided in nominal size <2 mm, and it was ground to <0.5 mm at each facility. For the in situ CFP, the feed was additionally sieved using a mesh 40 screen (420 μm) to remove dust. The elemental analyses performed at each site separately showed that the feed contained 50.7 ± 0.9 wt % carbon, 6.2 ± 0.4 wt % hydrogen, 0.11 ± 0.08 wt % nitrogen, 43.0 ± 1.2 wt % oxygen, and 0.5 ± 0.2 wt % inorganics on dry biomass basis.

The base zeolite catalyst was ZSM-5 extrudates from Zeolyst (CBV 8014) with a silica-to-alumina molar ratio of 80. The catalyst was ground and sieved, and particles of 300–1000 μm were used in the experiments. The catalyst was presteamed ex situ at 600 °C for 30 min to add mesoporosity to the catalyst. The presteamed catalyst Brunauer–Emmett–Teller (BET) surface area was 339 m^2^/g by nitrogen physisorption, and acid site density was 519 μmol/g by NH_3_ temperature-programmed desorption. The acid sites consisted of 369 μmol/g of Brønsted acid sites by isopropyl amine temperature-programmed desorption and 150 μmol/g of Lewis acid sites by difference.

A catalyst with 2.4 wt % Pt on Evonik-Aerolyst 7711 TiO_2_ support was utilized for the fixed-bed ex situ CFP experiments.^2^ The catalyst was prepared using standard incipient wetness techniques.^2^ The catalyst was reduced ex situ at 450 °C in flowing 5 vol % H_2_ in N_2_ for 2 h (5 °C/min heating rate) and passivated at room temperature in flowing 1 vol % O_2_ in N_2_ for 8 h prior to the CFP experiments. The support pellet diameter was 1.6–1.7 mm, and the bulk density was 0.95–1.12 g/cm^3^. The BET surface area was 44 m^2^/g, and the pore volume was 0.30–0.45 cm^3^/g. The acid site density by NH_3_ temperature-programmed desorption was 220 μmol/g, and metal site density by CO chemisorption was 40 μmol/g.^2^

The catalyst for the in situ CFP experiments was produced from red mud supplied by Almatis, Inc. (Burnside, LA) and was formulated with alumina and silica as binders and attrition-resistant components to reduce losses in the fluidized-bed reactor.^22^ The losses were less than 2 wt % during 24 h of continuous fluidization at twice the minimum fluidization velocity. The catalyst had a particle size range of 250–600 μm and was calcined at 550 °C in a muffle furnace for 5 h before being used. The fresh catalyst had a surface area of 78 m^2^/g and a pore volume of 0.30 cm^3^/g.

The hydrotreating catalyst was a commercial supported Ni–Mo catalyst ground and sieved to size 0.25–0.60 mm.^22^ The catalyst bulk density was 0.47 g/cm^3^. The catalyst was sulfided in situ prior to each hydrotreating experiment.^2^

### CFP Experiments

The CFP reactor systems and operating procedures have been described in detail in previous publications,^2,22,42^ and a summary of the operating conditions is shown in Table 1. Briefly, the ex situ zeolite CFP utilized two fluidized-bed reactors, one for pyrolysis and one for upgrading,^42^ and the ex situ HDO catalyst CFP used the same fluidized-bed pyrolysis reactor connected to a fixed-bed upgrading reactor.^2^ One fluidized-bed reactor with a batch of catalyst was employed for the in situ CFP.^22^ The CFP catalysts and operating conditions were selected separately for each process. The pyrolysis temperature was the same for the two ex situ experiments (500 °C) but lower (400 °C) for the in situ experiment, in which pyrolysis and upgrading were performed in the same reactor. The upgrading temperature was 500 °C for the zeolite catalyst and 400 °C for the HDO (Pt/TiO_2_) catalyst. Temperatures were chosen because prior experiments had suggested these to give the best CFP performance.^22,38^ Hydrogen was included as the feed gas for the HDO catalyst to enable the HDO reactions, whereas nitrogen was utilized for the zeolite catalyst.

**Table 1:** Operating Conditions for the CFP Experiments

catalyst type	zeolite	HDO	mixed metal oxide
catalyst	HZSM-5	Pt/TiO_2_	red mud
mode	ex situ	ex situ	in situ
pyrolysis reactor	fluidized bed	fluidized bed	fluidized bed
upgrading reactor	fluidized bed	fixed bed	
pyrolysis temperature, °C	500	500	400
pressure	atmospheric	atmospheric	atmospheric
biomass feed rate, g/h	420	150	1000
feed gas	100% N_2_	85% H_2_/15% N_2_	15% N_2_/85% recycle gas
feed gas rate, sL/min	17.4	17.6	73
ex situ upgrading temperature, °C	500	400	
catalyst feed	continuous	batch	batch
catalyst mass or feed rate	420 g/h	100 g	1000 g
biomass fed/catalyst, g/g	1.4	2.8	10
catalyst regeneration	no	coke oxidation + catalyst reduction	no

Catalyst was continuously fed and removed in the ex situ zeolite CFP experiment in a once-through configuration without catalyst regeneration, whereas a constant batch of catalyst was utilized for the ex situ HDO catalyst and the in situ experiments. The zeolite CFP oil was produced with a 1.4 g/g ratio of the biomass feed to the catalyst feed. The HDO catalyst CFP oil was produced over 13 cycles of catalytic upgrading and regeneration.^2^ Each catalytic cycle was continued until the mass of biomass fed over the catalyst mass (biomass/catalyst) reached a predetermined value (2.8 g/g), after which coke was removed from the catalyst by oxidation in a mixture of air and nitrogen, and the catalyst was subsequently reduced in hydrogen prior to the beginning of the next catalytic cycle. No increases in oil oxygen content or reductions in oil carbon yield were evident over the 13 experiments, and irreversible catalyst deactivation was negligible.^2^ The in situ CFP oil was collected during a total of 10 h of operation over 2 days without catalyst regeneration. As shown in Figure S1, the in situ CFP oils produced at different time-on-stream (TOS) values had very similar elemental compositions, but the CFP oil viscosity increased, indicating some deactivation of the catalyst over time.

### Hydrotreating

A bench-scale hydrotreater configured as a single-pass, cocurrent, continuous, down-flow reactor was used for the CFP oil hydrotreating tests. The hydrotreater is described in detail in previous publications.^2,10^ Approximately 20 mL of the 0.25–0.6 mm catalyst was placed in the isothermal zone, and 6 mL of the catalyst extrudates on top of that in the temperature transition zone. The catalyst was presulfided in situ by 35 wt % di-*tert*-butyl disulfide (DTBDS) in decane. The operating pressure was 13.0 MPa, maximum temperature 400 °C, and the sulfiding agent liquid hourly space velocity (LHSV) was 0.12 L/(L of catalyst h) during presulfidation.

DTBDS was added to the CFP oil feeds to maintain 150 ppm of S in the CFP oil in order to retain the catalyst in the sulfided form. The isothermal hydrotreating temperature was 400 ± 3 °C, the pressure 12.8 ± 0.5 MPa, and the H_2_/CFP oil ratio 2700 ± 50 (sL H_2_)/(L CFP oil). 80–84 h tests at LHSV of 0.20 (L CFP oil)/(L catalyst h) were conducted with all CFP oils and an additional 22–60 h at LHSV of 0.30 (L CFP oil)/(L catalyst h).

### Oil Analyses

CFP and hydrotreated oils were analyzed for CHN by ASTM D5291/D5373, for O by ASTM D5373mod, and for S by ASTM D1552/D4239, and for water content by Karl Fischer titration (ASTM D6869). The CFP oils and hydrotreated products were analyzed by gas chromatography–mass spectrometry (GC–MS) and carbon nuclear magnetic resonance ^(13^C NMR) as described previously.^2,43^ A modified ASTM standard method D664 for determining the acid content of petroleum products was used to determine carboxylic acid numbers (CANs).^44^

### Oil Fractionation and Fuel Property Analysis

Fractions boiling in the gasoline and diesel range were separated from the hydrotreated oils by distillation in a B/R 800 micro spinning band distillation system equipped with a metal band with 14 theoretical plates. Lights boiling below 70 °C were separated by atmospheric distillation and higher boiling fractions by vacuum distillation at 658 Pa (5 Torr). The fractions with atmospheric equivalent temperatures (AETs) below 182 °C were classified as the gasoline range, and those with AETs of 182–330 °C were classified as the diesel range. The material remaining in the flask was called residual, and losses (4–6% of the input mass) were attributed to material remaining in the column and the lines after distillation and were included in the diesel-range yield. The gasoline-range fraction was characterized by detailed hydrocarbon analysis (DHA) according to ASTM D6729. The diesel-range fractions were analyzed for the derived cetane number (DCN) according to ASTM method D6890.

## Results and Discussion

### Catalytic Fast Pyrolysis

CFP was performed for each process type (ex situ CFP with a zeolite catalyst, ex situ CFP with an HDO catalyst, and in situ CFP with a red mud catalyst) at conditions summarized in Table 1. The zeolite catalyst produced oil with 17 wt % oxygen on a dry basis at a carbon efficiency of 28% (Table 2). The carbon yield lies within the range of those reported (24–30%) for CFP oils with similar oxygen contents for zeolite-based CFP in either in situ or ex situ configuration at the bench or pilot scale.^15,16,45^ The HDO catalyst delivered a similar deoxygenation performance as the zeolite catalyst did but at a higher oil carbon efficiency (38%). The higher carbon efficiency is attributed to the presence of hydrogen and the HDO activity of the catalyst, which reduces coke formation via hydrogenation of coke precursors and increases carbon retention in the oil.^2^ The carbon yield in coke for the HDO catalyst was half of that for the zeolite catalyst (6% vs 13%).

**Table 2:** Product Mass and Carbon Yields during CFP and CFP Oil Compositions. Results for Ex Situ Fixed Bed from Ref (2)

	ex situ zeolite	ex situ HDO	in situ red mud
yield, g/g dry biomass			
oil	19%	27%	30%
aqueous	23%	25%	18%
gases	29%	30%	31%^a^
char	11%	10%	21%^b^
coke	7%	3%	
total	90%	94%	100%^a^
C yield, g C/g C in biomass			
oil	28%	38%	37%
aqueous	3%	3%	5%
gases	24%	26%	24%
char	17%	16%	22%^b^
coke	13%	6%	
total	86%	88%	89%
oil composition and H/C ratio			
C, wt % dry basis	74.6%	74.9%	65.1%
H, wt % dry basis	6.6%	7.6%	6.8%
N, wt % dry basis	0.1%	0.2%	0.1%
O, wt % dry basis	17.3%	17.3%	28.1%
H_2_O, wt %	3.4%	4.7%	5.3%
H/C, mol/mol	1.06	1.23	1.24
H/C_eff_ = (H–2O)/C, mol/mol	0.72	0.90	0.61
CAN, mg KOH/g	54	35	63

a Gases for in situ are by difference.

b Char + coke for in situ.

The in situ CFP over red mud retained a similar fraction of biomass carbon in the CFP oil (37%) as the HDO catalyst did, but the oil had a significantly higher oxygen content (28 wt %). Characterization of the spent red mud catalysts and activity measurement of oxidatively regenerated catalysts showed that although inorganics (Ca, K, Mg, and P) from biomass were deposited on the catalyst, they had a minimal negative impact on catalyst activity.^23^ However, the red mud catalyst presented a poorer deoxygenation performance because of its less active basic sites and the lower CFP temperature used (400 °C vs 500 °C in the ex situ systems). Char and coke were not separated in the in situ CFP with the red mud catalyst; however, the limited deactivation observed for this catalyst over 10 h suggests that coke formation may have been low. The high char yield for in situ CFP can be related to the low pyrolysis temperature.

The largest loss of carbon in all systems was to light gases (24–26%, Table 2), and the produced gases consisted of carbon oxides with lower fractions of light hydrocarbons (Table 3). CO and CO_2_ formation was high for the in situ CFP, which suggests that decarbonylation and decarboxylation were more significant deoxygenation routes than dehydration or HDO for this catalyst. This is supported by the lower aqueous phase mass yield for this catalyst (Table 2). The high carbon dioxide formation by red mud may be related to the ketonization activity of the basic sites in red mud, e.g., Fe_2_O_3_.^1^

**Table 3:** Mass and Carbon Yields of Gases during CFP

	ex situ zeolite		ex situ HDO		in situ red mud	
gas yields	mass	C	mass	C	mass	C
H_2_	0.1%				0.1%	
CO	15.6%	13.4%	14.5%	12.6%	11.5%	10.0%
CO_2_	8.2%	4.5%	8.2%	4.5%	16.7%	9.2%
CH_4_	0.9%	1.8%	2.3%	3.5%	0.6%	0.9%
C_2_–C_5_	3.4%	5.9%	5.3%	6.0%	2.3%	4.0%

Carbon losses to the aqueous phase were low in all systems (≤5%, Table 2), indicating that the CFP organic products had low solubility in water due to the reduced polarity of the molecules compared to the polarity of the molecules in noncatalytic fast pyrolysis oils.

The zeolite CFP oil had the lowest hydrogen-to-carbon (H/C) molar ratio (Table 2), indicative of the high aromatic content typical for CFP oils upgraded over zeolites.^1,2,5^ The HDO and the red mud CFP oils had similar H/C ratios, but the effective H/C ratio was higher for the HDO oil. The effective H/C is defined as (mol H – 2 × mol H)/(mol C) and reflects the H/C if all remaining oxygen is removed as water^46,47^ and could be used as a better measure than direct H/C for predicting hydrogen requirement during hydrotreating. The CANs varied between 35 and 63 mg of KOH/g (Table 2), and the CAN was highest for the red mud CFP oil. As a comparison, CAN for noncatalytic fast pyrolysis of pine has been reported to be 76.^15^

**Figure 1:**
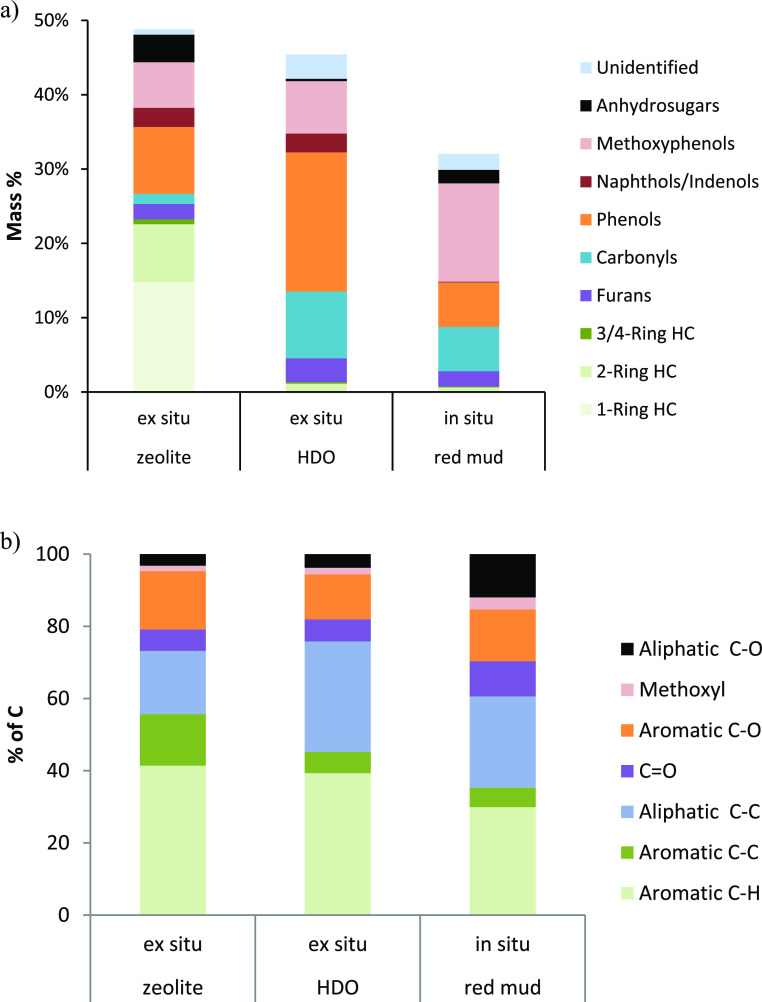
(a) GC–MS and (b) ^13^C NMR analysis of CFP oils. HCs refers to hydrocarbons, and phenols refer to phenolics without methoxy functionalities.

There were significant differences in the compositions of the CFP oils. The GC–MS-detectable portion constituted 32–49% of the CFP oil mass (Figure 1a). The nondetected portion can be mainly attributed to high-boiling compounds, such as lignin oligomers, though water and low-boiling compounds covered by the solvent also contribute to the undetected fraction. The non-GS–MS-detectable fraction was the highest for the red mud CFP oil, consistent with the lower degree of upgrading for this catalyst. The zeolite CFP oil contained high fractions of fully deoxygenated aromatic hydrocarbons (23 wt % in the oil) and, in addition, both partially upgraded oxygenates, such as phenols without additional oxygen functional groups, indenols and naphthols, furans, and carbonyls, and primary pyrolysis vapor compounds, such as methoxyphenols and anhydrosugars (levoglucosan). In contrast, the GC–MS-detectable compounds in the HDO CFP oil consisted mainly of partially deoxygenated vapors (phenols and carbonyls as the largest group) with some primary vapors (methoxyphenols, though negligible anhydrosugars). The GC–MS-detectable fraction of the red mud CFP oil had the largest content of primary vapors (methoxyphenols and anhydrosugars).

Phenols without methoxy groups were the largest detected oxygenate group in both the zeolite and HDO CFP oils, but there were some differences in the compounds (Table S1). Phenols in the HDO oil often included the original side chains in the lignin monomers, e.g., ethyl and propyl groups, whereas the phenols in the zeolite CFP oil, likely formed via a phenol pool mechanism,^48^ contained mainly methyl side chains. The zeolite CFP oil also included significant concentrations of benzenediols, but these were present at lower concentrations in the HDO CFP oil. Benzenediols were also prevalent within the phenols for the red mud CFP oil. Naphthols and indenols were present in both the zeolite and HDO CFP oils but were mostly absent from the red mud CFP oil. The red mud CFP oil, on the other hand, had the highest methoxyphenol content.

The phenols and methoxyphenols mainly stem from the lignin fraction of the biomass, whereas the carbohydrate fraction forms carbonyls, furans, anhydrosugars, and aromatic hydrocarbons. Aromatic hydrocarbons were prevalent only in the zeolite oil, with toluene and xylenes being the most prominent compounds (Figure 1a and Table S1). Carbonyls were prevalent in the HDO and red mud CFP oils; cyclopentenones were the main detected carbonyl group for all CFP oils, the HDO oil also included cyclopentanones, and the red mud oil contained hydroxyketones (e.g., hydroxypropanone). 2-Carbonylfurans (e.g., furfural and 5-methyl-2-furancarboxaldehyde) were dominant in the zeolite and red mud CFP oils, whereas the furans in the zeolite oil consisted mainly of benzofurans.

Unlike GC–MS, ^13^C NMR can detect the carbon bonds in all organic compounds. The results (Figure 1b and Table S2) agree well with the general trends seen by the GC–MS analysis. Total aromatic carbon (aromatic C–C, C–H, and C–O), which includes carbons in the rings of aromatic hydrocarbons, phenolics, and higher hydroaromatic compounds as well as those in furan rings,^43^ constituted at least 50% of the carbon for all three CFP oils. The aromatic fraction was highest for the zeolite oil, consistent with the highest content (48 wt %) of GC–MS groups related to these carbon types (aromatic hydrocarbons, phenols, methoxyphenols, indenols, naphthols, and furans) and lowest for the red mud oil, which contained only 22 wt % material detected in these compound groups by GC–MS. Aliphatic C–O bonds originate in the carbohydrate fraction, and they were present at the highest concentration in the red mud CFP oil. This contributes not only to the high oil yield for this pathway but also to the high oxygen content of the oil. The aliphatic C–C includes side chains in other compound groups, e.g., aromatic hydrocarbons and phenols. These chains were longer in the HDO oil than those in the zeolite CFP oil, and ^13^C NMR also suggested a higher content of them in the HDO than in the zeolite CFP oil (31% vs 18% of carbon). The higher aliphatic C–C content in the HDO than that in the zeolite CFP oil explains that the two oils had similar oxygen contents even though the zeolite oil had a significant proportion of aromatic hydrocarbons, whereas the HDO oil contained very little hydrocarbons. In fact, the ^13^C NMR results suggest a slightly lower total O/C ratio (fraction of aromatic and aliphatic C–O bonds) for the HDO than that for the zeolite CFP oil. The O/C ratio is highest for the red mud CFP oil, in agreement with its highest oxygen content.

In summary, CFP with the HDO catalyst required hydrogen addition but produced a CFP oil with a low oxygen content (17%) at a high carbon efficiency (38%). CFP with the zeolite catalyst produced oil with a similarly low oxygen content but at a lower carbon efficiency (28%), while CFP with red mud gave a high carbon efficiency (37%), but the oil oxygen content was also high (28 wt %). The zeolite catalyst, which contained a high density of Brønsted acid sites, favored aromatic hydrocarbon and phenol formation, whereas the HDO catalyst, with both acid and metallic hydrogen-activating sites and with added hydrogen gas, favored phenol and cyclopentenone formation. The red mud catalyst, which contains low densities of both acid and base sites,^23^ retained the highest fractions of primary pyrolysis vapors, such as methoxyphenols and high-molecular-weight material, not detectable by GC–MS.

Inorganic impurities in biomass impact catalyst deactivation and, thus, the catalyst lifetime in the CFP process. Catalyst deactivation and accumulation of metals have been discussed for each of the processes in previous publications. For HZSM-5, K accumulation was found to occur throughout the catalyst particles, including within the catalyst pores leading to decreased catalyst activity and changes in catalyst functionality, potentially via formation of basic metal sites.^7^ Other metals, such as Mg and Ca, deposited only on the outer surface of the catalyst particles. For the Pt/TiO_2_ catalyst, sintering of the Pt particles was observed after 13 regeneration cycles, leading to an increase in Pt particle size with a concomitant decrease in metal sites, whereas the acid site densities remained relatively constant and only small variations were observed in the catalyst activity.^2^ Via simulated accumulation of K on Pt/TiO_2_, K impacted mainly acid-catalyzed alcohol dehydration at low K loadings, whereas at high loadings (<800 ppm on catalyst), K poisoned the interfacial active sites for HDO and CO oxidation reactions.^49^ For the red mud catalyst, metal accumulation and catalyst deactivation were investigated during eight regeneration cycles.^23^ K accumulation was linear with the number of regenerations, whereas the accumulation of Ca and Mg leveled off after a couple of regeneration cycles and was attributed to chemical reactions with alumina to form aluminates. The number of both acid and basic sites decreased in regenerated catalysts; however, the CFP oil yield was not impacted by catalyst regeneration though there was evidence of increased cracking of high-molecular-weight compounds, likely due to the impact of K. Due to the basic sites in red mud, K did not negatively impact the red mud catalyst activity.

Inorganic components derived from the biomass or the catalysts may also negatively impact hydrotreating either by poisoning the hydrotreating catalyst active sites or otherwise depositing in the catalyst bed. The inorganic content is expected to be lowest in the HDO CFP oil because of the ex situ configuration and the use of a fixed bed. Due to the brittle nature of the red mud catalyst, compared to the zeolite catalyst, it may have the highest potential for catalyst fines carryover to the CFP oil. Filtering of the CFP oils to remove particulates may be necessary.

### Hydrotreating of the CFP Oils

The CFP oils were hydrotreated over a commercial NiMo sulfide catalyst using a continuous trickle-bed reactor for 82–84 h at an LHSV of 0.2 h^–1^ after which the LHSV was increased to 0.3 h^–1^ for 22–60 h. The LHSV impacts the hydrotreating cost,^50^ and the LHSV was increased to determine if the higher LHSV was sufficient to deoxygenate the CFP oils. No bed plugging was experienced in any hydrotreating experiment, and the experiments were terminated when all feed was consumed. Table 5 summarizes the hydrotreating results with an LHSV of 0.2 h^–1^, and more details at both LHSV’s can be found in Figure S2 and Table S3. The oil carbon yields at 0.2 h^–1^ LHSV ranged from 81 to 93% and increased in the order of red mud < zeolite < HDO CFP oil. The light gas formation was the main source of carbon losses. The formation of all light gas groups (carbon oxides, methane, and C_2_–C_5_ hydrocarbons) was highest for red mud oil (Table 5). The red mud CFP oil retained the highest fractions of oxygenated compounds such as acids, methoxy phenols, and low-molecular-weight carbonyls that led to the formation of carbon oxides, methane, and C_2_–C_5_ hydrocarbons, respectively.

**Table 4:** Summary of Hydrotreating Results at 0.2 h^–1^ LHSV

	ex situ zeolite	ex situ HDO	in situ red mud
yield, g/g dry CFP oil			
oil	83%	76%	61%
water	14%	19%	30%
gases	4.1%	6.5%	15%
total	96%	98%	99%
C yield, g C/g C in CFP oil			
oil	93%	89%	81%
gases	3.6%	6.5%	15%
total	96%	96%	96%
average product oil composition			
O, wt % dry basis	0.05%	0.2%	0.9%
H/C, mol/mol dry basis	1.55	1.71	1.66
H_2_ consumption			
g/g dry CFP oil			
hydrotreating	0.053	0.039	0.059
CFP		0.033	
total, g/g dry CFP oil	0.053	0.072	0.059
g/g hydrotreated product			
hydrotreating	0.064	0.051	0.097
CFP		0.043	
total, g/g hydrotreated product	0.064	0.094	0.097
CFP + hydrotreating C efficiency			
g C/g C in biomass	26%	34%	30%

**Table 5:** Gas Yields during Hydrotreating at 0.2 h^–1^ LHSV

gas yield, g/g dry CFP oil	ex situ zeolite	ex situ HDO	in situ red mud
CO	0.2%	0.3%	0.6%
CO_2_	0.8%	1.3%	3.0%
CH_4_	0.4%	0.6%	1.3%
C_2_–C_5_	2.7%	4.3%	9.5%

Good deoxygenation was obtained for both the zeolite and HDO CFP oil (Table 4), which had similar low oxygen contents (Table 2). For the hydrotreated red mud CFP oil, the average oxygen content was 0.9%, but the oxygen content of the product continuously increased as a function of TOS and exceeded 1 wt % at the last measurement point at 0.2 h^–1^ LHSV (Table S3). Successful one-stage hydrotreating is important for the economics of fuel production via CFP processes.^17^ The result suggests, therefore, that CFP oil oxygen content needs to be lower than that of the red mud CFP oil here (28 wt %) or the hydrotreating conditions need to be more severe (lower LHSV, higher temperature, or hydrogen partial pressure) and/or several hydrotreating stages in increasing severity are required to enable more stable hydrotreating and low oxygen content in the hydrotreated fuel product. Catalyst deactivation rate during hydrotreating of CFP oils has been found to correlate with the total oxygen content in CFP oil,^51^ but the upper limit for oxygen is likely dependent on the oxygenated species, and the impact of different oxygen compound groups on hydrotreating performance needs to be established.

The product quality decreased as the LHSV was increased (Table S3); the oil oxygen content increased and exceeded the target of <1 wt % oxygen, the product H/C decreased, the density increased, and the products contained more high-boiling material (Table S4). These indicate lower deoxygenation, hydrogenation, and cracking efficiencies during hydrotreating at the higher space velocity and suggest that the LHSV needs to be <0.3 h^–1^ for all CFP oils tested.

The high CFP carbon efficiency for the HDO CFP oil, combined with a relatively low oxygen content in the CFP oil, resulted in the highest overall carbon efficiency from biomass to hydrotreated products (34%, Table 4). The zeolite pathway gave the lowest overall carbon efficiency (26%) due to the low CFP carbon efficiency, despite the highest hydrotreating efficiency. The red mud CFP oil had a high CFP carbon efficiency, similar to that of the HDO CFP oil, but the red mud CFP oil retained a high oxygen content and a variety of oxygenated groups, which leads to significant losses during hydrotreating, and it gave a medium overall carbon efficiency (30%). Noncatalytic fast pyrolysis followed by hydroprocessing has been reported to give higher overall carbon efficiencies of 41–43%,^52^ but the poor quality of noncatalytic fast pyrolysis oil makes hydrotreating challenging, necessitating several hydrotreating stages.^39,40,50^

Hydrogen consumption is an important performance matrix because of the cost of hydrogen production.^53^ The hydrogen consumption per gram of CFP oil feed during hydrotreating was lowest for the HDO CFP oil (0.39 g/g of CFP oil, Table 4). This CFP oil had a low oxygen content and the highest effective H/C ratio (Table 2), and therefore less hydrogen was needed to process it during hydrotreating. However, hydrogen was also consumed during the CFP process for this pathway, and the overall hydrogen consumption per g of CFP oil was the highest (0.072 g/g CFP oil) for this pathway. The CFP and hydrotreating yields varied between the pathways, and another important matrix is how much hydrogen is required per g of the final product.^54^ On this basis, the hydrogen consumption during hydrotreating was highest for the red mud CFP oil (Table 4) due to the high amount of CFP oil that needs to be processed to produce an equivalent amount of hydrotreated product. Per gram of product, the hydrogen consumption during hydrotreating was lowest for the HDO pathway; however, if one takes into account hydrogen consumption during CFP, the total hydrogen consumption for the HDO pathway was similar to that for the red mud pathway. For the overall hydrogen consumption per gram of product, the zeolite pathway had the lowest hydrogen consumption, which can be attributed to the low CFP and high hydrotreating yield.

Overall, the HDO pathway gave the highest product carbon yield at the expense of the highest overall hydrogen consumption per gram of hydrotreated oil produced. The process, however, allowed 46% of the hydrogen to be provided at a close to atmospheric pressure during the CFP step. The zeolite pathway gave the lowest hydrogen consumption and the lowest overall product yield.

The stable operation of a single-stage hydrotreater for more than 100 h without catalyst bed plugging issues represents a much improved bio-oil processability compared to that of noncatalytic pyrolysis oil, which normally leads to rapid reactor plugging during its hydrotreating within 60 h.^39^ It suggests that CFP could enable the production of stable bio-oil and, therefore, eliminate bio-oil stabilization processes required for noncatalytic bio-oil, such as low-temperature hydrogenation, which is costly and challenging. However, product quality deterioration as a function of TOS was observed for processing the red mud CFP oil, indicative of catalyst deactivation. Further longer-term hydrotreating tests should be conducted with CFP oils from all pathways to demonstrate the long-term processability of these CFP oils and to obtain correlations of processability with CFP bio-oil properties. The validation of the stability and processability of the CFP oils is critical for the success of the CFP process.

Per GC–MS analysis (Figure S3 and Table S5), the hydrotreated products consisted mainly of cyclic compounds, either fully aromatic (e.g., alkylbenzenes), partially hydrogenated (e.g., tetrahydronaphthalenes), or completely saturated cycloalkanes (e.g., cyclohexanes and decahydronaphthalenes). Over half of one-ring compounds (55–80%) were cycloalkanes, and the majority (56–72%) of the identified 2- and 3-ring compounds were partially hydrogenated and had retained one aromatic ring. Some straight-chain alkanes, e.g., pentane, were also present in particular in the hydrotreated HDO and red mud CFP oil products. The oxygenates remaining were mainly phenols, and their concentrations were highest in the product from red mud oil, consistent with the highest hydrotreated oil oxygen content. No oxygenates were detected in the product from the zeolite oil. ^13^C NMR analysis (Figure S4) identified a high fraction of aliphatic C–C bonds in all oils (68–77%) with the remaining being aromatic (23–32%). The fraction of aromatic compounds was highest for the zeolite product as determined by both GC–MS and ^13^C NMR.

The hydrotreated products were fractionated to obtain products boiling in the gasoline and diesel ranges. The ex situ zeolite pathway gave the highest fraction (89 wt %) of material boiling in the fuel (gasoline + diesel) range, and the in situ red mud pathway gave the lowest fraction (81 wt %) (Table 6). Additional hydrocracking and/or recycle of the residue to the hydrotreater may be required to eliminate the residues.^55^ The ex situ products had higher fractions boiling in the gasoline range than in the diesel range; in contrast, the in situ process produced more diesel than gasoline fraction, reflecting the lower cracking activity during CFP for this process.

**Table 6:** Mass Yields from Fractionation of Hydrotreated Oils and Properties of the Fuel Fractions

	ex situ zeolite	ex situ HDO	in situ red mud
LHSV during hydrotreating, h^–1^	0.2	0.2	0.2 + 0.3
oil O, wt % dry basis	0.05	0.2	∼2.0
fraction, mass %			
gasoline range (25–182 °C AET^a^)	50%	45%	37%
diesel range (182–∼320 °C AET^a^)	39%	39%	43%
residual	11%	16%	19%
gasoline + diesel range	89%	84%	81%
gasoline range RON^a^	83	67	59
gasoline range MON^a^	72	62	55
AKI (RON + MON)/2^a^	77	64	57
diesel range DCN^a^	13	24	26

a AET = atmospheric equivalent temperature, RON = research octane number, MON = motor octane number, AKI = antiknock index = (RON + MON)/2, DCN = derived cetane number.

By DHA (Table S6), all of the fractions boiling in the gasoline range had high naphthenic (cycloalkane) contents (49–66 wt %), consistent with the GC–MS analysis results for one-ring compounds. Naphthenic rings have low octane numbers, and consequently, all oils had low octane numbers, antiknock indexes (AKI, average of research and motor octane numbers, RON and MON) of 57–77 (Table 6). In contrast to saturated ring structures, aromatics have high octane numbers, and the product octane numbers were highest for the ex situ zeolite pathway product, which had the highest aromatic content (Figures S6 and S7) and lowest for the in situ red mud pathway product, which had the lowest aromatic content. The octane number could be improved by reducing hydrogenation to retain aromatic rings; this could produce a gasoline blendstock of higher quality. Naphthenic compounds, on the other hand, are an important component in jet fuel, whose boiling point overlaps with that of both gasoline and diesel, and the hydrotreated products could be good candidates for sustainable aviation fuel.^56,57^

The diesel-boiling fractions had DCN of 13–26, all below the US minimum finished diesel fuel value of 40. The value was the lowest for the ex situ zeolite product, which had the highest aromatic content (Figures S6 and S7). Aromatics, which were present in all of the hydrotreated oils, do not have good autoignition properties. Enhanced hydrogenation of the rings and potentially opening of the ring structures would be required to improve the quality of the diesel-range product. Gasoline and diesel fractions have different requirements for producing high-quality fuel blendstocks, and a more complicated hydrotreating scheme than utilized here is required, for example, first-stage hydrotreating with low hydrogenation, separation of the gasoline-range product, and a second-stage hydrotreating to hydrogenate the diesel fraction.

## Conclusions

In this study, we compared the performance of three CFP pathways from feedstock to fuel blendstocks using the same southern pine feed: (1) ex situ CFP over a zeolite-based catalyst, (2) ex situ CFP over an HDO (Pt/TiO_2_) catalyst, and (3) in situ CFP over a red mud catalyst. The CFP conditions were selected separately for each pathway, but all of the CFP oils were hydrotreated in the same reactor under identical conditions.

Ex situ CFP with the HDO catalyst gave the highest carbon yield to hydrotreated product (34%) due to high CFP efficiency and good CFP oil properties. However, this pathway requires hydrogen addition during the CFP process, which adds complexity to the CFP process and increases cost, in particular, if CFP and hydrotreating are performed at different locations. The addition of hydrogen together with solid biomass also poses safety challenges.

In situ CFP with the red mud catalyst gave the second highest carbon yield to the hydrotreated product (30%) and offers the possibility for a less costly CFP process. The potential challenge with this pathway lies in the poorer quality of the CFP oil, which resembles noncatalytic fast pyrolysis oil. The results here indicated some deactivation during hydrotreating and suggest that more severe hydrotreating conditions or a more complicated hydrotreating scheme than tested here is required for stable operation.

Ex situ CFP with the zeolite catalyst gave the lowest carbon efficiency, but it is the most technologically mature pathway, and CFP over zeolite catalysts is being commercialized in both in situ and ex situ configurations for the production of chemicals (benzene, toluene, and xylene) by companies such as Anellotech and BioBTX.^58^

Advances in the hydrotreatment process are required to improve the fuel blendstock properties for all pathways. The ex situ zeolite pathway gave a gasoline-range product with the highest quality due to the highest aromatic content of this product, and the in situ red mud pathway gave the best quality for a diesel-range product. However, the gasoline- and diesel-range fractions for all processes failed to meet octane and cetane number requirements. To improve the gasoline-range product properties, limiting excessive hydrogenation to retain aromatics would be one option. To improve the diesel-range product quality, saturation of the aromatic rings combined with the opening of the ring structures prevalent in the oils would be helpful. Aviation fuel encompasses both aromatic and naphthenic hydrocarbons, and the suitability of the products for sustainable aviation fuel should be explored.

In order for CFP with hydrotreating to become a viable pathway to produce biofuels, improvements are required in both the CFP and hydrotreating processes to improve yield and product quality. Both processes need to be scaled up, and the robustness of the processes needs to be demonstrated by experiments of longer duration.

## Supporting Information Available

Bio-oil composition as a function of time for CFP over red mud, GC–MS and ^13^C NMR analytical results for bio-oils and hydrotreated products, detailed hydrotreating results, hydrocarbon analysis summary for gasoline fractions (PDF)
