# Supplementary material for: From Biomass to Fuel Blendstocks via Catalytic Fast Pyrolysis and Hydrotreating: An Evaluation of Carbon Efficiency and Fuel Properties for Three Pathways
Source: Energy Fuels. 2023 Nov 29;37(24):19653–63. doi: 10.1021/acs.energyfuels.3c03239 (PMC10749445; doi:10.1021/acs.energyfuels.3c03239)
Supplement: Supplementary file 1 — ef3c03239_si_001.pdf [file ef3c03239_si_001.pdf]

From biomass to fuel blendstocks via catalytic fast pyrolysis and hydrotreating: an evaluation of carbon efficiency and fuel properties for three pathways

Kristiina Iisa[1*], Calvin Mukarakate[1], Richard J. French[1], Foster A. Agblevor[2], Daniel M. Santosa[3], Huamin Wang[3*], A. Nolan Wilson[1], Earl Christensen[1], Michael B. Griffin[1], Joshua A. Schaidle[1]

[1]National Renewable Energy Laboratory, Golden, CO 80403, USA

[2]Utah State University, Logan, UT 84322, USA

[3]Pacific Northwest National Laboratory, Richland, WA 99354, USA

* Corresponding authors: kristiina.iisa@nrel.gov, huamin.wang@pnnl.gov

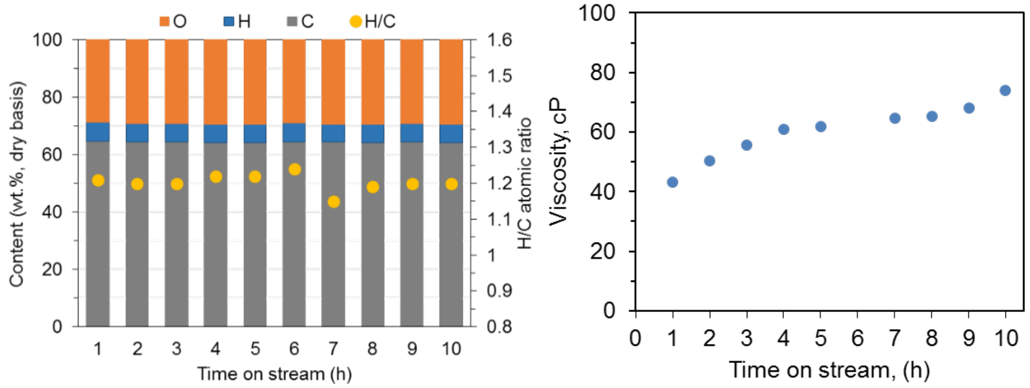

Figure S1. Composition and viscosity of bio-oil produced at different time-on-stream for CFP of pine using red mud catalyst in the *in situ* CFP.

Table S1. Major compounds detected in CFP oils at concentrations ≥0.5 wt% by GC-MS; however, the data in Figure 1a is based on all detected compounds. The assignments are based on matches to NIST library and have not been verified.

Compounds, wt%	ex situ zeolite	ex situ HDO	in situ red mud
Benzene	0.6%		
2-Propanone, 1-hydroxy-			0.9%
Toluene	4.7%		
Ethylbenzene	0.7%		
Cyclopentanone		1.1%	
p-Xylene	3.4%		
m-Xylene	2.4%		
o-Xylene	1.0%		
Furfural		0.6%	0.9%
2-Cyclopenten-1-one	0.9%	2.1%	0.5%
Benzene, 1-ethyl-2-methyl-	0.5%		
Benzene, 1,2,3-trimethyl-	0.8%		
2-Cyclopenten-1-one, 2-methyl-		1.3%	
Phenol	1.7%	3.1%	
2-Furancarboxaldehyde, 5-methyl-		0.6%	0.5%
Indane	0.7%		
2-Cyclopenten-1-one, 3-methyl-		2.2%	0.5%
2-Cyclopenten-1-one, 3-methyl-			0.6%
Indene	1.0%		
Phenol, 2-methyl-	1.3%	1.4%	0.5%
2-Cyclopenten-1-one, 2-hydroxy-3-methyl-			1.4%
Phenol, 3/4-methyl	1.8%	2.4%	0.5%
Benzofuran, 2-methyl-	0.8%		
Phenol, 2-methoxy-	0.6%	0.6%	1.2%
Cycloprop[a]indene, 1,1a,6,6a-tetrahydro-	0.7%		
Phenol, 2,5-dimethyl-	0.8%	0.9%	
Phenol, 3,5-dimethyl-	0.5%		
Phenol, 4-ethyl-		0.9%	0.5%
Phenol, 3-ethyl-		1.6%	
Naphthalene	0.9%		
2-methoxy-4-methylphenol	0.9%	0.8%	2.3%

1,2-Benzenediol	0.9%		1.1%
Phenol, 2-ethyl-5-methyl-		0.7%	
Propylphenol (mixture)		2.0%	
Phenol, 3-ethyl-5-methyl-		0.7%	
Phenol, 4-ethyl-2-methoxy-			1.3%
1,2-Benzenediol, 4-methyl-	0.6%		
Naphthalene, 2-methyl-	1.8%		
1,2-Benzenediol, 4-methyl-	0.5%		0.7%
Phenol, 4-(1-methylpropyl)-		0.7%	
Unknown			0.5%
2-Methoxy-4-vinylphenol		1.1%	
Benzaldehyde, ethyl-		0.6%	
Phenol, 2-methoxy-4-propyl-			0.9%
Phenol, 4-(2-propenyl)-		1.1%	
Phenol, 2-methoxy-3-(2-propenyl)-		1.0%	1.1%
Unknown		1.2%	
Naphthalene, 1,4-dimethyl-	0.9%		
1H-Indenol	0.7%	0.5%	
Phenol, 2-methoxy-4-(1-propenyl)-		0.4%	0.9%
Phenol, 2-methoxy-4-(1-propenyl)-	1.0%	1.6%	2.8%
Benzaldehyde, 3-hydroxy-4-methoxy-	0.9%		
Vanillin	0.9%	0.5%	0.7%
Apocynin	0.6%		
2-Naphthalenol	0.6%		
Levoglucosan	3.7%		1.6%
Benzeneacetic acid, 4-hydroxy-3-methoxy-			0.8%

Table S2. ^{13}C NMR analysis of CFP and hydrotreated (HT) oils.

		Ex situ riser		*Ex situ* fixed bed		*In situ* fluid. bed	
	ppm shift	CFP	HT	CFP	HT	CFP	HT
C=O	215-166.5	5.9	0.0	6.1	0.0	9.7	0.0
Aromatic C-O	166.5-142	16.2	3.0	12.5	5.6	14.4	6.1
Aromatic C-C	142-132	14.2	7.6	5.9	5.6	5.4	4.8
Aromatic C-H	132-95.8	41.4	21.7	39.3	15.3	29.9	12.2
Aliphatic C-O	95.8-60.8	3.3	0.0	3.8	0.0	12.0	0.0
Methoxyl	60.8-55.2	1.5	0.0	1.8	0.0	3.3	0.5
Aliphatic C-C	55.2-0	17.6	67.7	30.6	73.5	25.3	76.5

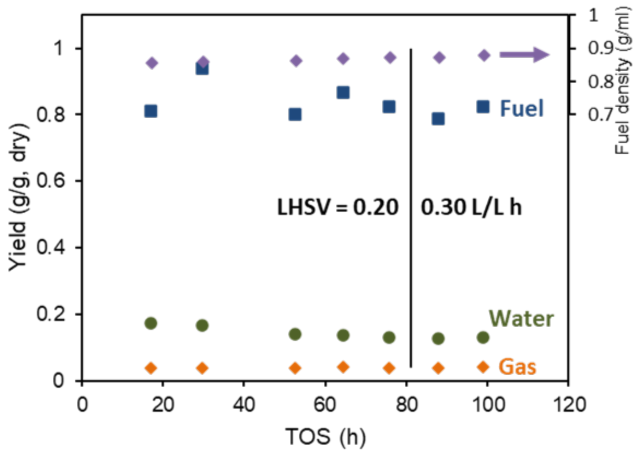

a)

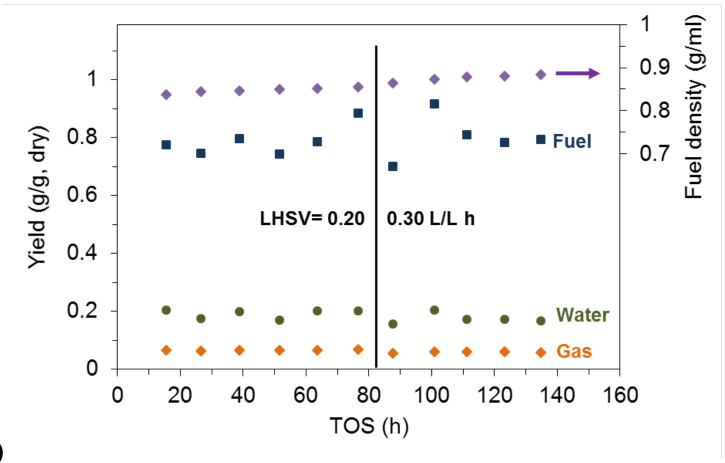

b)

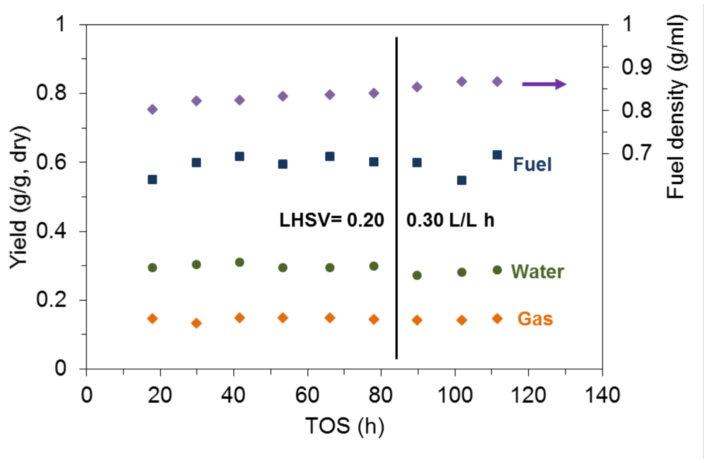

c)

Figure S2. Yield of hydrotreated oil product (fuel), gas, and water product and density of fuel product at different time on stream (TOS) for a) *ex situ* zeolite CFP oil, b) *ex situ* HDO CFP oil, and c) *in situ* red mud CFP oil.

Table S3. Detailed yield, carbon yield, H_2 consumption, and mass balances of selected samples for hydrotreating. Data from *ex situ* fixed-bed reactor CFP oil at 0.2 L9/(L h) is from Griffin et al., *Energy & Environmental Science,* DOI: 10.1039/c8ee01872c

Sample TOS (h)	Fuel product Yield (w/w, dry)	Carbon yield (w/w)	H/C (mol/mol)	O (wt.%, dry)	Density (g/ml)	Gas product Yield (w/w dry)	Carbon yield (w/w)	Produced Water Yield (w/w dry)	H_2 Consumption (w/w dry bio-oil)	Mass Balance (%)	Carbon Balance (%)
***Ex situ* zeolite CFP oil, LHSV 0.2 h^{-1}**											
45-60	0.80	0.90	1.57	NA*	0.862	0.040	0.036	0.14	0.054	93.6	93.2
60-69	0.87	0.97	1.57	NA*	0.870	0.041	0.037	0.14	0.053	99.4	100.7
69-82	0.83	0.92	1.57	NA*	0.871	0.040	0.036	0.13	0.053	94.9	95.9
**Average**	**0.83**	**0.93**	**1.57**	**0.05***	**0.870**	**0.040**	**0.036**	**0.14**	**0.053**	**96.0**	**96.6**
***Ex situ* zeolite CFP oil, LHSV 0.3 h^{-1}**											
93-104	0.83	0.92	1.50	1.5	0.878	0.0410	0.035	0.131	0.051	95.2	95.9
***Ex situ* HDO CFP oil, LHSV 0.2 h^{-1}**											
45-58	0.74	0.87	1.68	0.28	0.850	0.065	0.065	0.17	0.034	94.7	93.2
58-69	0.79	0.91	1.74	0.10	0.852	0.065	0.065	0.20	0.043	100.8	97.9
**Average**	**0.76**	**0.89**	**1.71**	**0.19**	**0.851**	**0.065**	**0.065**	**0.19**	**0.039**	**97.8**	**95.5**
***Ex situ* HDO CFP oil, LHSV 0.3 h^{-1}**											
116-130	0.78	0.90	1.60	1.82	0.880	0.060	0.061	0.17	0.036	98.4	96.7
***In situ* red mud CFP oil, LHSV 0.2 h^{-1}**											
24-36	0.60	0.81	1.63	0.65	0.823	0.13	0.14	0.30	0.061	97.9	94.9
48-60	0.60	0.79	1.70	0.95	0.833	0.15	0.16	0.30	0.056	98.5	94.7
72-84	0.62	0.82	1.66	1.21	0.842	0.15	0.15	0.30	0.059	100.2	97.1
**Average**	**0.61**	**0.81**	**1.66**	**0.93**	**0.833**	**0.15**	**0.15**	**0.30**	**0.059**	**98.9**	**95.6**
***In situ* red mud CFP oil, LHSV 0.3 h^{-1}**											
96-115	0.62	0.82	1.62	2.70	0.868	0.15	0.15	0.29	0.065	99.2	96.0

* Individual data not available by direct O measurement; only average for the combined product measured.

Table S4. Calculated weight distributions of different fractions of hydrotreating products based on simulated distillation results. QC is an internal standard composite of diesel #2 obtained from retail stations.

Sample	Gasoline, wt.%	Jet A, wt.%	Diesel, wt.%	Residual, wt.%
TOS (h)	(<184 ºC)	(153-256 ºC)	(184-344 ºC)	(>344 ºC)
QC Diesel	10.7	42.0	77.8	11.5
Ex situ zeolite, LHSV 0.2 h^{-1}				
45-58	49.8	32.0	40.6	9.6
58-69	48.4	31.9	41.6	10.0
Ex situ HDO, LHSV 0.2 h^{-1}				
45-58	45.6	28.9	39.0	15.5
58-69	46.9	28.3	38.2	14.8
Ex situ HDO, LHSV 0.3 h^{-1}				
116-130	38.6	30.4	43.4	18.0
In situ red mud, LHSV 0.2 h^{-1}				
48-60	46.0	27.6	38.8	15.2
72-84	43.3	28.4	40.5	16.2
In situ red mud, LHSV 0.3 h^{-1}				
96-115	38.0	29.5	44.7	17.4

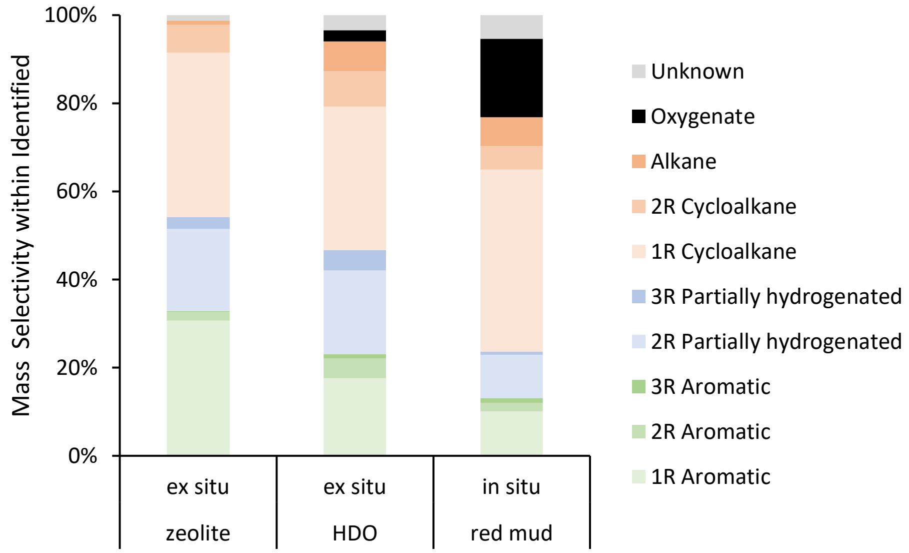

Figure S3. GC-MS analysis of hydrotreated oils. The *ex situ* zeolite and HDO oils are composites from LHSV 0.2 h^{-1} but the *in situ* oil includes products from both LHSV of 0.2 and 0.3 h^{-1}.

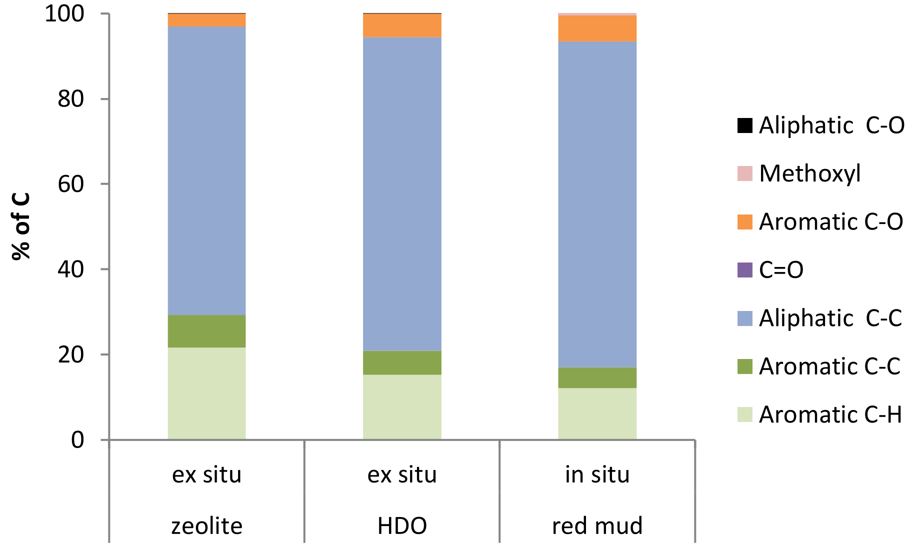

Figure S4. ^{13}C NMR analysis of hydrotreated products. The *ex situ* zeolite and HDO oils are composites from LHSV 0.2 h^{-1} but the *in situ* oil includes products from both LHSV of 0.2 and 0.3 h^{-1}.

Table S5. Major compounds detected in the hydrotreated oils at concentrations ≥0.5 wt% by GC-MS. The results in Figure 3 are based on all detected compounds but only compounds >0.5wt% are included here. The assignments are based on matches to NIST library and have not been verified. The *ex situ* zeolite and HDO oils are composites from LHSV 0.2 h^{-1} but the *in situ* oil includes products from both LHSV of 0.2 and 0.3 h^{-1}.

Compound, wt%	ex situ zeolite	ex situ HDO	in situ red mud
Pentane		1.0%	
Cyclopentane, methyl-	0.8%		
Cyclohexane	3.7%		
Cyclopentane, ethyl-			4.4%
Cyclohexane, methyl-	6.4%		
Benzene	0.5%		0.6%
Cyclohexane, dimethyl-	1.5%	0.8%	1.5%
Cyclohexane, dimethyl-	0.5%		0.5%
Cyclohexane, dimethyl-	1.1%	0.5%	0.7%
Cyclopentane, propyl-			1.0%
Cyclohexane, ethyl-	3.1%	2.7%	3.8%
Toluene	5.3%	1.2%	1.3%
Nonane			0.7%
Cyclohexane, trimethyl-		0.9%	0.9%
Cyclohexane, ethyl-methyl	0.9%		
Cyclohexane, ethyl-methyl	0.7%	0.8%	1.0%
Cyclohexane, ethyl-methyl			0.8%
Cyclohexane, propyl-	2.9%	3.2%	5.7%
Ethylbenzene	1.4%	0.7%	0.5%
p/m-Xylene	3.3%	0.7%	0.6%
m-Xylene	3.0%		
Cyclohexane, ethyl-methyl-			0.5%
Cyclohexane, methyl-propyl-	0.6%	0.5%	
Decane			0.9%
Benzene, dimethyl-	1.4%		
Cyclohexane,ethyl-dimethyl-			0.8%
Cyclohexane, methyl-propyl-			0.6%
1H-Indene, octahydro-, trans	0.5%		
1H-Indene, octahydro-, cis	2.0%	1.2%	0.9%
Benzene, propyl-	0.7%	1.0%	0.9%
Cyclohexane, butyl-			0.6%
Benzene, ethyl-methyl-	0.6%	0.6%	
Benzene, ethyl-methyl-	0.7%		
1H-Indene, octahydro-, methyl-	0.6%		
Benzene, trimethyl-	1.0%	0.5%	
Unknown			0.5%
Benzene, methyl-(methylethyl)-			0.9%
Naphthalene, decahydro-	0.6%		
Phenol			1.1%

Naphthalene, decahydro-methyl-		0.5%	0.6%
Indane	2.4%	1.0%	0.6%
Dodecane			0.6%
Naphthalene, decahydro-methyl-			0.6%
Phenol, methyl-			0.8%
Indane, methyl-	0.7%		
Indane, methyl-	0.9%		
Benzene, methyl-(methylethyl)-		0.5%	
Phenol, methyl-			1.0%
1H-Indene, 2,3-dihydro-4-methyl-		0.5%	0.6%
Naphthalene, 1,2,3,4-tetrahydro-	2.6%	0.5%	
Phenol, dimethyl-			0.8%
Phenol, ethyl-			0.9%
Phenol, ethyl-methyl-			0.9%
Naphthalene, 1,2,3,4-tetrahydro-methyl-	1.1%	0.6%	
Propylphenol (mixture)			1.4%
Naphthalene, 1,2,3,4-tetrahydro-methyl-	2.0%		
Naphthalene, 1,2,3,4-tetrahydro-dimethyl-	1.0%		
Naphthalene, 1,2,3,4-tetrahydro-trimethyl-			0.7%
Pentadecane			0.9%
Phenol, (methylpropyl)-			1.3%
1H-Fluorene, dodecahydro-			0.6%
Unknown			1.1%
Naphthalene, dimethyl-		0.6%	0.6%
Unknown			0.6%
Hexadecane			1.1%
Unknown			0.7%
1H-Indene, 2,3-dihydro-trimethyl-		0.6%	
1H-Indene, 2,3-dihydro-(methylpropyl)-			0.7%
Unknown		0.6%	0.6%
Octahydrophenanthrene, isopropyl-trimethyl-		0.5%	

Table S6. Detailed hydrocarbon analysis for gasoline fractions.

GROUP for PIANO ANALYSIS, vol%	*Ex situ* zeolite	*Ex situ* HDO	*In situ* red mud
Paraffins	3.3	7.4	9.0
i-Paraffins	3.9	3.3	5.1
Aromatics	40.6	17.0	11.4
Mono-Aromatics	37.7	15.1	10.4
Naphthalenes	0.0	0.0	0.0
Naphtheno/Olefino-Benzenes	0.2	0.2	0.1
Indenes	2.7	1.6	0.9
Naphthenes	49.1	65.7	66.3
Mono-Naphthenes	48.9	65.3	65.5
Di/Bicyclo-Naphthenes	0.2	0.4	0.7
Olefins	0.2	0.1	0.4
n-Olefins	0.0	0.0	0.0
Iso-Olefins	0.2	0.0	0.1
Naphtheno-Olefins	0.0	0.1	0.3
Di-Olefins	0.0	0.0	0.0
Oxygenates	0.1	0.7	0.3
Unidentified	2.8	5.8	7.5
OCTANE NUMBERS			
RON	74	67	59
MON	69	62	55
(RON+MON)/2	71	65	57
Vapor Pressure (psi)	1.8	2.6	2.8
